# Comparing the costs and outcomes of an integrated twin compression screw (ITCS) nail with standard of care using a single lag screw or a single helical blade cephalomedullary nail in patients with intertrochanteric hip fractures

**DOI:** 10.1186/s13018-018-0923-x

**Published:** 2018-08-30

**Authors:** Leo M. Nherera, Paul Trueman, Alan Horner, Alan J. Johnstone, Tracy J. Watson, Francis A. Fatoye

**Affiliations:** 1 Health Economics, Smith & Nephew Global Market Access, 101 Hessle Road, Hull, HU3 2BN UK; 2 University of Aberdeen and Aberdeen Royal Infirmary, Aberdeen, UK; 3 Orthopedic and Spine Institute at Banner University Medical Center, Phoenix, AZ USA; 4 Department of Health Professions, Manchester Metropolitan University, Manchester, UK

**Keywords:** Cephalomedullary nail, Intertrochanteric hip fractures, Cost-effectiveness analysis, Single lag screw, Single helical blade screw, Integrated twin compression screw

## Abstract

**Background:**

Surgical treatment is the optimal strategy for managing intertrochanteric fractures as it allows for early rehabilitation and functional recovery. The purpose of the study was to assess the cost-effectiveness of commonly used cephalomedullary nails for the treatment of unstable intertrochanteric hip fractures.

**Methods:**

A decision analytic model was developed from a US payer’s perspective using clinical data from a pairwise meta-analysis of randomised controlled trials (RCTs) and comparative observational studies comparing the integrated twin compression screw (ITCS) nail versus two single-screw or blade cephalomedullary nails [single lag screw (SLS) nail and single helical blade (SHB) nail]. The model considered a cohort of 1000 patients with a mean age of 76, as reported in the clinical studies over a 1-year time period. Cost data was obtained from the Center for Medicare and Medicaid Services website and published literature and adjusted for inflation. One-way and probabilistic sensitivity analyses were conducted to assess the effect of uncertainty in model parameters on model conclusions.

**Results:**

The model estimated 0.546 quality-adjusted life years (QALYs) and 0.78 complications avoided by using the ITCS nail and 0.455 QALYs and 0.67 complications avoided for the standard of care, using SLS or SHB nails. The cost per patient was $34,336 for patients treated with an ITCS nail and $37,036 for patients treated with the standard of care respectively, resulting in a cost saving of $2700 in favour of the ITCS nail. More savings were observed when the ITCS nail was compared to the SHB ($3280 per patient) and SLS ($1652 per patient). The findings were robust to a range of both one-way and the probabilistic sensitivity analyses.

**Conclusion:**

In conclusion, the ITCS nail can be considered a cost saving intervention in patients undergoing intertrochanteric fracture fixation with an intramedullary device. Clinicians and policy makers should be encouraged to adopt healthcare technologies such as ITCS that will help them to provide quality healthcare despite falling budgets.

## Background

Hip fractures are a major public health problem in terms of patient morbidity, mortality and costs to health and social care. The incidence of hip fractures increases exponentially with age with approximately 90% of hip fractures occurring in people older than 65 years [1] due to higher rates of osteoporosis and falls in the elderly population [2]. As the population ages, the number of fractures is estimated to increase from about 320,000 per year to 580,000 by 2040 in the USA with healthcare costs exceeding $10 billion per year [3–5] placing a huge financial burden on patients, families, insurers, hospitals and governments. Furthermore, in the USA, it is estimated that hip fractures account for approximately 14% of all fractures, but impart nearly 70% of the acute hospital care costs associated with fracture treatment [6]. The 1-year mortality for hip fractures ranges from 14 to 36%, with 30% more deaths observed than the age-matched population [4].

In 2010, in the European Union, there was an estimated incidence of 600,000 incident hip fractures costing €20 billion. In Germany, the incidence of hip fractures is estimated at 125,000 (152/100,000 inhabitants) cases per year and costs related to hip fractures are estimated to be 2.8 billion EURO per year [7]. In the UK, the incidence of hip fractures is estimated to be between 70,000 to 75,000 per year, the annual cost is estimated to be over £2 billion and the incidence is predicted to increase to 104,000 by 2025 [2, 8] leading to increasing pressure on the falling healthcare budgets.

Surgical intervention is deemed to be the definitive treatment for hip fractures as it allows for early patient mobilisation [9–11] and economic evaluations have concluded that surgery for hip fracture is cost saving when compared to non-surgical treatment [1]. Consequently, a variety of surgical techniques and implants have been introduced for the treatment of intertrochanteric fractures. In particular, the use of cephalomedullary nail fixation has been shown to deliver superior clinical outcomes relative to sliding hip screws in patients with unstable fractures [9–11]. With increasing pressures on healthcare budgets in many healthcare systems, it is important to have robust clinical and economic evidence on the performance of the different cephalomedullary fixation nails to assist clinical decision making and provide value to the patients and payers.

An economic evaluation estimated the cost-effectiveness of sliding hip screws compared with cephalomedullary nails for patients with intertrochanteric hip fractures and concluded that sliding hip screws were cost-effective in stable fractures while cephalomedullary nails were cost saving in unstable fractures [12]. The cost-effectiveness of cephalomedullary nails in unstable fractures is therefore established in patients with intertrochanteric hip fractures. However, it is unclear which among the cephalomedullary nailing systems is the best option in this patient population. Our study therefore aimed to compared three commonly used cephalomedullary fixation nails, the integrated twin compression screw (ITCS) nail (TRIGEN^◊^ INTERTAN Smith & Nephew, Memphis, TN, USA) compared with a single helical blade (SHB) nail (Proximal Femoral Nail Antirotation (PFNA™) DePuy Synthes, Solothurn, Switzerland) and a single lag screw (SLS) nail (Gamma3™; Stryker, Schönkirchen, Germany) in the treatment of intertrochanteric fractures.

## Methods

A decision analytic model was constructed in Microsoft Excel 2016 (Microsoft Corporation, Redmond, WA, USA) to estimate the expected total costs and health benefits expressed in quality-adjusted life years (QALYs) between cephalomedullary nails. The mean age of patients included in the model was 76 years as reported in the studies that were included in the meta-analysis. The model was assessed over a 1-year time period as the majority of the outcomes were reported at 1 year. The model is assessed from the US payer’s perspective in particular the Center for Medicare and Medicaid Services (CMS). We did not discount the costs and benefits since the time horizon was short and up to 1 year.

**Fig. 1:**
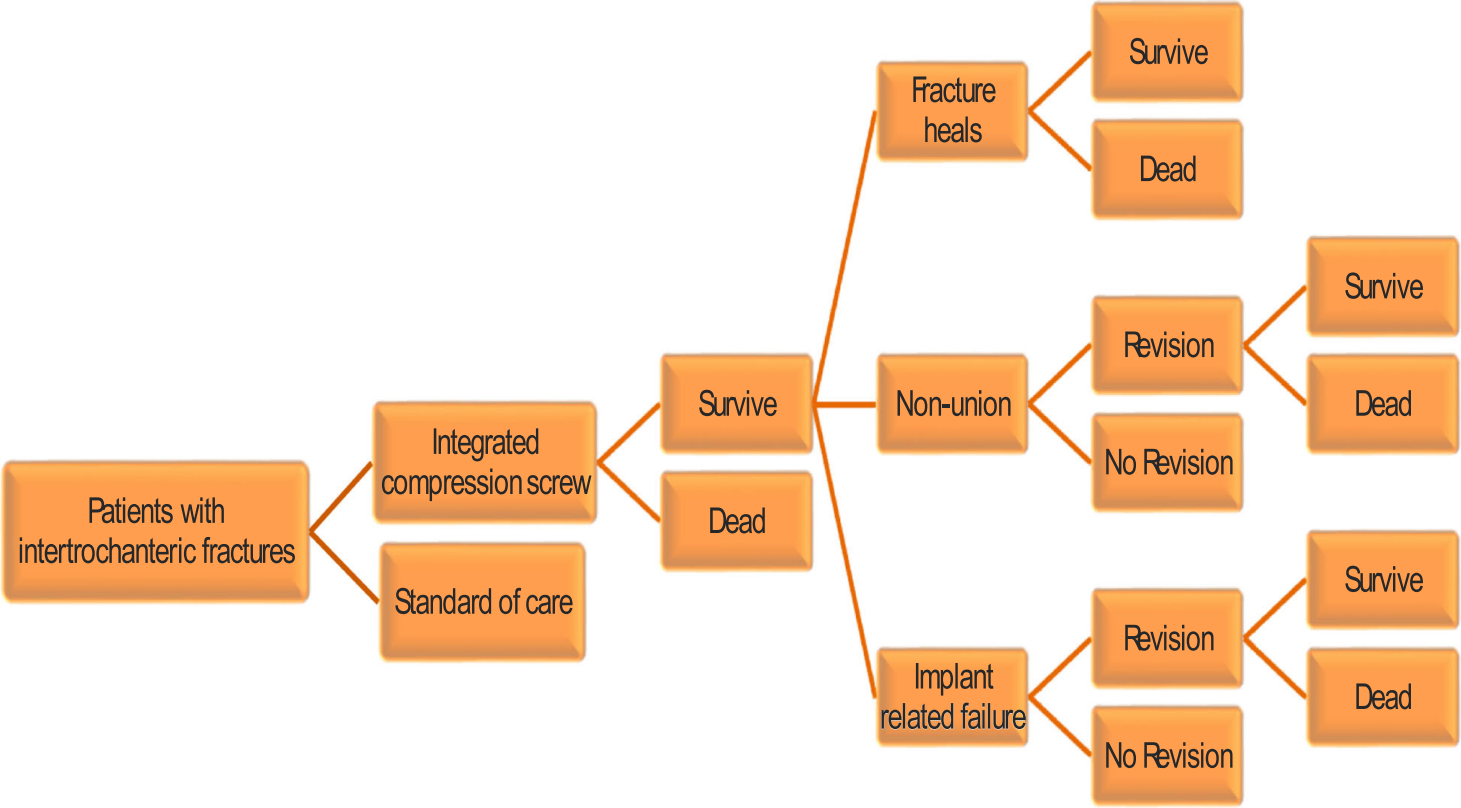
Integrated twin compression screw (ITCS) nail compared with standard of care using a single lag screw or a single helical blade; cost-effectiveness model structure

The outline of the decision tree used is shown in Fig. 1. The Figure shows the branches for the ITCS strategy, and there are similar branches for the standard of care strategy although these have not been included in the figure. The figure demonstrates that when patients enter into the model, they may develop complications or die. The modelled complications or health states are implant-related failures, non-unions, healed state and death. A proportion of patients who experience complications are further exposed to the risk of revision surgery to treat a complication which may or may not be successful. For the patients who require revision surgery, an additional risk of mortality associated with the revision surgery was assumed in accordance with published literature [12].

### Clinical parameters used in the economic model

Data for this economic analysis were derived from the individual meta-analysis comparing ITCS with SHB and SLS cephalomedullary nails. We updated a published meta-analysis that compared the ITCS with SHB nail [13] by including studies that compared ITCS with SLS nail [14–16]. Thus, the baseline data on complications, i.e., implant-related failures, non-unions and revisions, were taken from the event rates seen in the combined SHB and SLS nail arms of the meta-analysis. The ITCS nail treatment effect (odds ratios) was then applied in the model taken from the same meta-analysis.

All patients were at risk of age adjusted all-cause mortality which was obtained from US Life tables 2014 [17]. Hip surgery is also known to be associated with an increased risk of mortality, and therefore, the increase in mortality due to hip surgery was modelled using data which was obtained from a study by Swart et al. [12]. Additional mortality for patients who had revision surgery was included and taken from the same study by Swart [12]. We assumed that any mortality was independent of the implant nail used, and therefore, no treatment effect on mortality was applied. We adopted this approach because mortality is likely to be influenced by other factors such as comorbidities due to the age of these patients who are treated using these implants. All clinical data used in the model is shown in Table 1.

**Table 1:** Clinical parameters used in the economic model

Outcome	Mean	*N*	Events	No events	Source
Implant failures	0.159	762	121	641	Updated Nherera 2018 [13]
Revisions	0.071	637	45	592	
Non-unions	0.022	447	10	437	
Outcome	Mean	Lower 95 CI	Upper 95% CI	SE	Source
Mortality due to fractures	0.06	0.03	0.12	0.02	Swart 2014 [12]
Revision surgery mortality	0.06	0.0001	0.09	0.02	
Health-related quality of life (utility data)					
Utility for healed fracture	0.700	0.630	0.800	0.044	Swart 2014 [12]
Utility for revision	0.600	0.450	0.750	0.080	
Effectiveness of integrated compression screw					
Implant-related failures	0.15	0.09	0.24	0.25	Updated Nherera 2018 [13]
Revisions	0.31	0.17	0.56	0.30	
Non-unions	0.54	0.17	1.66	0.58	
Proportion of patients in nursing home					
Nursing home	0.24	0.18	0.3	0.03	Gu 2016 [1]

The lower and upper values for revision utility and proportion of patients in nursing home were assumed to be 25% below and above the reported mean values

*Abbreviations*: *CI* confidence interval, *SE* standard error, *N* total number of patients for the outcome

Rates of long-term nursing home care for patients following surgery were modelled using data from Gu et al. [1], who conducted an economic analysis of surgical compared to non-surgical treatment for displaced intracapsular and extracapsular hip fractures. Long-term nursing home care in the surgical arm was 16% for patients aged 65 to 74 years old, 24% for patients 75 to 84 years old, and 48% for patients older than 85 years. The model used 24% since the mean age of the cohort was 77 years.

### Cost data used in the economic model

Cost data used in the reference case, and the ranges used in sensitivity analysis are given in Table 2. The study used the following inpatient ICD-9 diagnosis codes 820.0x, 820.1x, 820.2x and 820.3x to identify patients with extracapsular fractures. The average of Center for Medicare and Medicaid Services Medicare Payments of Diagnostic Related Groups (DRGs) 461, 462, 469,470,480–81 were applied in the model. The costs of the cephalomedullary nail implants for the base case model were based on mean values reported in the Premier Database. The study by Swart [12] assumed no difference in implant costs, and we adopted the same assumption in this model. Costs of revision surgery, long-term nursing home and annual follow-up costs following discharge from hospital were obtained from a published study by Gu et al. [1] and were adjusted for medical inflation to 2017 USD. We assumed that follow-up costs were the same for both revision and primary operations. Furthermore, we did not explicitly model revision costs of a total hip replacement following implant failure. The proportion of patients that ended up in a nursing home was independent of the choice of the implant used as there was no specific data by implant.

**Table 2:** Cost data used in the model

Cost centre	Mean	Lower value	Upper value	Source
Cost of surgery	$18,058	$13,543	$22,572	CMS^a^
Cost of revision	$37,036	$24,691	$49,382	Swart 2014 [12]
Annual post discharge costs	$15,976	$7989	$18,259	Gu 2014 [1]
Costs of treating non-healed fractures	$652	$489	$815	CMS^a^
Nursing home	$91,971	$45,985	$137,956	Gu 2014 [1]
Cephalomedullary nail implant cost	$3000	$1500	$4500	2016 Premier^b^ database

The lower and upper cost values were assumed to be 50% above or below the reported mean cost

^a^CMS—Center for Medicare and Medicaid Services; https://www.cms.gov/

^b^ https://www.premierinc.com/wpdm-package/research/

### Health-related quality of life

The utility values which are used to estimate the quality-adjusted life years (QALYs) were obtained from a published economic evaluation by Swart [12]. Utility values are between 0.0 for death and 1.0 for best possible health. The reported utility scores for patients undergoing a successful initial fixation of an intertrochanteric fracture were reported to be 0.70 with lower and upper values of 0.63–0.80. The utility loss following revision surgery was estimated to be − 0.10 [12]. The utility values were age adjusted as there is evidence to suggest that health-related quality of life is negatively correlated with age [18]. Utility data used in the model are shown in Table 1.

### Sensitivity analysis and sub-group analysis

We conducted both one-way and probabilistic sensitivity analyses to address the uncertainty around base case results for an adult with a mean age of 76 years. One-way sensitivity analyses were carried out by varying one input parameter at a time, assigning a low and high value and then evaluating the impact of that variation upon the model results. Probabilistic sensitivity analysis entails specifying a distribution for each model parameter and then simultaneously selecting values at random from those distributions using Monte Carlo simulation. Data on the ranges used in sensitivity analysis are shown in Table 1 for clinical parameters and Table 2 for costs. We also conducted sub-group analyses, and in particular, we considered the comparators individually, i.e., single helical blade nail and single lag screw nail.

## Results

The base case analysis from the payer’s perspective demonstrated that the ITCS nail was associated with lower total mean costs per patient and improved clinical outcomes compared to patients in the standard of care (single helical blade and single lag screw) group. The use of the ITCS nail is therefore a dominant strategy, i.e., cheaper overall and results in better clinical outcomes. Table 3 show the results of the economic analysis. The estimated cost savings for the ITCS nail compared to standard of care is estimated to be $2700 per patient per year.

**Table 3:** Base case results

Intervention	Costs	Complications avoided	QALYs	Cost savings
Standard of care	$37,036	0.67	0.455	
Integrated compression screw	$34,336	0.78	0.546	$2700

*Abbreviation*: *QALYs* quality-adjusted life years

### Sensitivity analysis

**Fig. 2:**
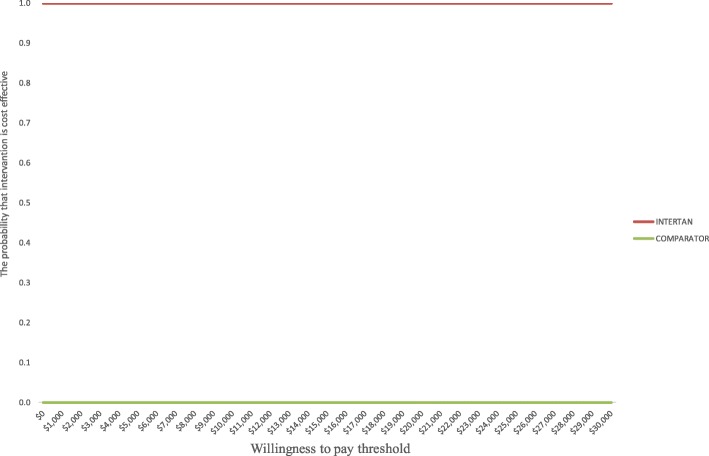
Integrated twin compression screw (ITCS) nail compared with standard of care using a single lag screw or a single helical blade; cost-effectiveness acceptability curves

**Fig. 3:**
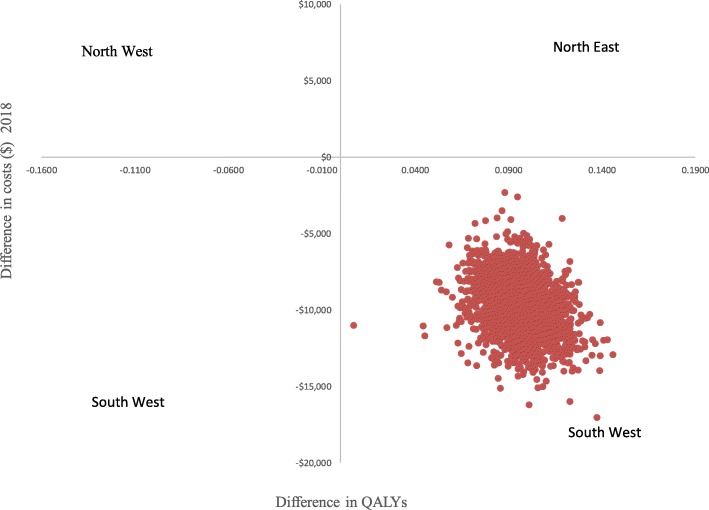
Integrated twin compression screw nail (ITCS) compared with standard of care using a single lag screw or a single helical blade; cost-effectiveness plane

A number of parameters were varied in the one-way sensitivity analysis shown in Table 4. The results of the one-way sensitivity analysis show that the ITCS nail remained cost saving when different assumptions were applied. In the base case model, we assumed that 24% of patients will end up in a nursing home and that all patients will incur $15,006 annual follow-up costs to capture the system costs. In sensitivity analysis, we assumed there were no follow-up costs including no nursing home costs to restrict the analysis to a hospital perspective. When this hospital perspective was adopted, ITCS remained cost saving, saving the hospital $414 per patient. Probabilistic sensitivity analysis demonstrated that the ITCS nail is 100% cost saving as shown by a flat cost-effectiveness acceptability curve at different willingness to pay values (Fig. 2) and Fig. 3 which is showing that all simulations are located in the south west quadrant.

**Table 4:** Sensitivity analysis: the ITCS nail compared to standard of care

Input parameter-mean (lower and upper value)	Savings when lower value is used	Savings when upper value is used
Effect on implant failures 0.31 (0.17–0.56)	$2883	$2427
Effect on revisions 0.15 (0.09–0.24)	$2708	$2687
Effect on non-unions 0.54 (0.17–1.66)	$2593	$3026
Mortality due to fractures 0.06 (0.03–0.12)	$3083	$1168
Revision surgery mortality 0.06 (0–0.09)	$2700	$2700
Proportion in nursing home 0.24 (0.18–0.30)	$2095	$4314
Cost of surgery $18,058 ($13,543–$22,572)	$2700	$2700
Cost of revision $37,036 ($27,777–$45,295)	$2620	$2781
Annual post discharge costs $15,976 ($11,982–$19,970)	$2734	$2667
Cost of implants $3000 ($1500–$4500)	$2684	$2717
Nursing home costs $91,971 ($45,985–$137956)	$1490	$3911
Removing nursing home and follow-up costs	$414

### Sub-group analysis

For sub-group analysis, we separately analysed the results of each of the single nail, SHB and SLS. Sub-group results for the single helical blade and the single lag screw all showed similar results to the combined results, see Tables 5 and 6. More complications were avoided when the ITCS nail was compared to the single helical blade than when compared to the single lag screw device. When compared to the single helical blade, the cost savings per patient was estimated to be $3280. In contrast, when compared to the single lag screw, the cost saving was $1652 per patient. Overall, the results of sensitivity and sub-group analyses were in agreement in that the ITCS nail always produced a cost-saving result and therefore remained dominant when compared to the standard of care.

**Table 5:** Sub-group analysis results: the ITCS nail compared to single lag screw nail

Intervention	Costs	Complications avoided	QALYs	Cost savings
Single lag screw	$36,024	0.69	0.476	
Integrated compression screw	$34,373	0.78	0.546	$1652

*Abbreviation*: *QALYs* quality-adjusted life years

**Table 6:** Sub-group analysis results: the ITCS nail compared to single helical blade nail

Intervention	Costs	Complications avoided	QALYs	1Cost savings
Single helical blade screw	$37,735	0.65	0.442	
Integrated compression screw	$34,455	0.78	0.543	$3280

*Abbreviation*: *QALYs* quality-adjusted life years

## Discussion

A number of surgical implants have been introduced for the treatment of intertrochanteric fractures, including cephalomedullary nails which have the benefit of allowing early mobilisation and minimise the risk of morbidity and mortality [3, 4]. Our study assessed the clinical and cost-effectiveness of the ITCS nail compared to standard of care, i.e., single lag screw nail or single helical blade nail use in patients with intertrochanteric fractures. This economic analysis demonstrated that the ITCS nail has advantages when compared to standard of care, offering fewer complications as measured by implant-related failures and revisions, but also cost savings of $2700 per patient from the payer’s perspective. These results were tested in sensitivity analysis and remained robust under various assumptions even when follow-up and nursing home costs were removed from the analysis, saving the hospital $414 per patient. Furthermore, the probabilistic sensitivity analysis increased our confidence in these findings with a 100% probability that the ITCS nail was cost saving at a threshold of $50,000/QALY.

We are not aware of any published cost-effectiveness studies that have compared different cephalomedullary nail fixation devices, although economic studies have been performed comparing cephalomedullary nail fixation with sliding hip screws. These studies found that cephalomedullary nail fixation was cost-effective for unstable fractures [12]. Our study has gone a step further and compared different cephalomedullary fixation nails making it the first paper of its kind to compare the ITCS nail to standard of care in this patient population.

Furthermore, our study is supported by robust clinical evidence based on a meta-analysis that included both observational and randomised controlled trials. The use of both observational and randomised controlled trials (RCTs) ensures that no useful evidence is discarded; thus, all available evidence is incorporated into the analysis. The single helical blade comparison had been evaluated in six studies, two RCTs [19, 20] and four [21–24] observational studies while the single lag screw analysis was evaluated in three studies, two RCTs [14, 15] and one observational study [16].

Our model made a number of conservative assumptions. We assumed revision was done with the same implant, and we did not explicitly model the progression of patients to total hip arthroplasty (THA). In reality, it is possible that patients who need a reoperation to treat failed internal fixation may end up with a conversion to a THA within the first year. This information on the conversion rates is not well characterised in the literature in relation to cephalomedullary nails in this population. This assumption is therefore biased against the implant with better revision rates, and the model may need updating once such information becomes more readily available. There are some subtle distinctions between the implants such as the amount of bone loss due to large entry portals in the greater trochanter which have not been captured in the model. However, these small differences are unlikely to result in a change of the overall model conclusions as the potential impact of these procedure-related events are already captured within the reported clinical outcomes for the nails.

Our analysis also assumed that all of the cephalomedullary nails had an equal impact on the proportion of patients who ended up in a nursing home. This assumption is conservative and favours the implants with a higher proportion of implant failure rates. The higher the proportion of implant failure, the more chances of requiring revision operations, which in turn may increase the chances of dependency. In this case, we may have underestimated the full potential savings of integrated twin compression screw nail which had superior revision and implant-related failure rates. This assertion was confirmed in sensitivity analysis when it was assumed that more patients in the standard of care group needed nursing home care. We noted more savings as opposed to the base assumption ($4314 vs $2700).

Furthermore, the model assumed only a 1 year time frame, and this may underestimate the long-term total benefits and costs; in particular, the studies were only able to capture revision rates at 1 year. There is a possibility of revision or even re-revision beyond 1 year which will affect the long-term costs and health-related quality of life. Literature demonstrates that the incidence of revision is higher in patients who have had one revision compared to those that have not failed. In this case, if the time intervals were extended, potentially more benefits would be seen for the intervention with better revision rates. However, we cannot quantify this benefit due to the lack of information in the published studies that were included in the analysis.

This study was carried out from the perspective of the US healthcare payer’s system and used average Medicare reimbursement as a proxy for the hospital costs. We wish to highlight two issues with this approach. Firstly, we are aware that there are some patients who do not fall under Medicare, and, secondly, the US costs may not be applicable in other healthcare systems. In the case of Medicare patients, there is a possibility that the total costs are underestimated, as Medicare usually negotiates lower costs than other payers, while the use of US costs tend to overestimate savings in other healthcare jurisdictions. We therefore suggest caution when interpreting these results in other healthcare systems, and encourage the use of local costs to test the robustness of our findings on a local basis. We suggest that the economic analysis should also be performed using different perspectives such as the societal to capture all possible long-term costs and benefits.

## Conclusion

In addition to other factors such as fracture type, pre- and postoperative morbidity of the patient and individual skills of the surgeon, this economic evaluation will assist decision makers to make choices that will optimise outcomes while accounting for differences in costs and uncertainty about the different interventions assessed. The analysis supports the use of the ITCS nail compared to standard of care (single helical blade and single lag screw nails in managing patients with unstable intertrochanteric fractures). These findings remained robust when different assumptions were tested. Studies with a longer time horizon maybe needed to confirm if the benefits persist beyond the reported 1 year and also include outcomes of possible conversion to total hip replacement for patients whose implants fail at 1 year. Clinicians and policy makers should be encouraged to adopt healthcare technologies such as ITCS that will help them to provide quality healthcare despite falling budgets.

## Abbreviations

CI: Confidence interval
CMS: Center for Medicare and Medicaid Services
ITCS: Integrated twin compression screw
PFNA: Proximal femoral nail anti-rotation
QALY: Quality-adjusted life years
RCT: Randomised controlled trial
SE: Standard error
SHB: Single helical blade
SLS: Single lag screw
THA: Total hip arthroplasty
